# Processing of Unattended Emotional Facial Expressions: Correlates of Visual Field Bias in Women

**DOI:** 10.3389/fnins.2017.00443

**Published:** 2017-08-14

**Authors:** Dina Wittfoth, Christine Preibisch, Heinrich Lanfermann

**Affiliations:** ^1^Institut für Diagnostische und Interventionelle Neuroradiologie, Medizinische Hochschule Hannover Hannover, Germany; ^2^Abteilung für Neuroradiologie, Klinikum rechts der Isar der TU München München, Germany

**Keywords:** visual hemifield presentation, emotion, emotional face processing, fearful face, happy face, functional magnetic resonance imaging

## Abstract

Lateralization in emotional processing is a matter of ongoing debate. Various factors can influence lateralized emotional processing, including stimulus location, emotional valence, and gender. In the present study, we aim to elucidate how unattended emotional facial expressions shown at different locations in the visual field influence behavioral responses, eye movement, and neural responses in a sample of healthy women. Our female participants viewed fearful, happy and neutral faces presented at central and peripheral (left or right) locations while keeping their gaze locked on a central fixation crosshairs and indicating stimulus location via button presses. Throughout the experiment, we monitored fixation and gaze shifts by means of eye tracking. We analyzed eye movements, neural and behavioral responses from *n* = 18 participants with excellent tracking and task performance. Face stimuli presented in the left hemifield entailed the fastest reactions irrespective of face valence. Unwarranted gaze shifts away from central fixation were rare and mainly directed at peripherally presented stimuli. A distributed neural network comprising the right amygdala, left temporal pole, left middle temporal gyrus, right superior frontal gyrus, and right posterior putamen differentially responded to centrally presented fearful faces, and to peripherally presented neutral and happy faces, especially when they appeared in the left hemifield. Our findings point to a visual field bias on the behavioral and neural level in our female sample. Reaction times, eye movements and neural activations varied according to stimulus location. An interactive effect of face location with face valence was present at the neural level but did not translate to behavioral or eye movement responses.

## Introduction

On a general level, the ability to discern relevant from irrelevant information entering one's sensory system is a crucial skill for any living organism. In humans, there is a large body of literature suggesting that the processing of task-relevant stimuli is prioritized over the processing of task-irrelevant stimuli (Lavie, 2005, 2010), while emotional stimuli tend to be attention-capturing cues even if they are not task-relevant (see also Pessoa, 2008). In fact, fear-related responses mediated by the amygdala might even be hard-wired and take place irrespective of attentional resources (Morawetz et al., 2010). Selective attention is capable of enhancing the processing of attended relative to unattended information. The enhanced processing of emotionally significant stimuli seems to rely on two attentional mechanisms: (i) “automatic” processing including a preattentive evaluation of emotional significance and (ii) “elaborate” processing preferentially allotting attentional resources to relevant stimuli.

The amygdala responds to unattended fearful faces presented peripherally both under low (Vuilleumier et al., 2001) and high (Williams et al., 2005) attentional load. Unattended fearful faces presented centrally elicit amygdala activation only under low attentional load (Anderson et al., 2003; Pessoa et al., 2005), but not high attentional load (Pessoa et al., 2002, 2005). The amygdala is one of the neural substrates of an enhanced processing mechanism for emotionally significant stimuli as it creates coarse, rapid, and automatic responses to emotional cues based on inputs from subcortical pathways (e.g., via the superior colliculus and pulvinar). A more fine-grained perceptual evaluation subsequently takes place e.g., in the occipital and temporal visual cortices (LeDoux, 2000, 2007; Adolphs, 2002; Phan et al., 2002).

Behavioral evidence for a visual field bias in emotional processing started to emerge about three decades ago. Split-field studies in healthy subjects pointed to the existence of a left visual field superiority in the perception of emotional facial expressions (Ley and Bryden, 1979; Strauss and Moscovitch, 1981; Mandal and Singh, 1990). These findings were matched by results from brain-damaged patients with lesions in the right hemisphere who showed significantly greater impairments in the identification and/or recognition of emotional facial expressions (Cicone et al., 1980; Borod et al., 1986; Mandal et al., 1991).

Results from various studies using brain imaging techniques corroborated and expanded these findings: a hemispheric bias for face processing in general was found in the right hemisphere in regions such as the occipital and the fusiform face area (Kanwisher and Yovel, 2006). Moreover, the perceptual discrimination of emotional faces from neutral faces was better if faces with positive valence were presented in the right hemifield and faces with negative valence were presented in the left hemifield (Jansari et al., 2000). Sung et al. (2011) suggest that a static circuit similar to passive information filtering is responsible for the involvement of low-level visual areas. Depending on the task, higher areas might dynamically modulate these neural circuits, as a right hemispheric (i.e., left visual field) superiority for facial processing differs with respect to stimulus content such as the orientation of faces in the picture (upright or inverted).

Previous work from our group also finds evidence for a visual field bias of neural activation in response to potential threat signals in a group of male subjects (Preibisch et al., 2009). The left lateral amygdala, bilateral inferior and middle frontal gyrus, left insula, right anterior cingulate cortex (ACC) and dorsomedial prefrontal cortex (dmPFC) as well as the bilateral superior temporal gyrus (temporal pole) responded to centrally presented neutral faces and to peripherally presented fearful faces, especially when they appeared in the right hemifield.

In the present combined eye tracking and functional imaging study, our aim was (i) to replicate the previously described visual field bias for peripherally presented fearful faces and centrally presented neutral faces in our sample of healthy females, and (ii) to compare these findings across stimulus valences by including faces with a positive emotional expression. Extending the approach of our previous study (Preibisch et al., 2009) we used concomitant eye tracking not only for monitoring task compliance but also for evaluating whether gaze deviations show a pattern of visual field bias.

## Materials and methods

### Participants

Twenty-eight right-handed healthy female subjects participated in the study after exclusion of acute or past neurological, psychiatric, or endocrine illness and use of psychotropic/endocrine medication. This study was carried out in accordance with the recommendations of Hannover Medical School's Ethics Committee with written informed consent from all subjects. All subjects gave written informed consent in accordance with the Declaration of Helsinki. The protocol was approved by the internal Ethics Committee of Hannover Medical School. We analyzed functional imaging data, eye tracking data and behavioral data from 18 participants (mean age (±*SD*) 24.8 ± 3.5 years) after excluding 10 participants due to either technical problems during functional imaging (*n* = 2), above cut-off depression scores (>14 in the Beck Depression Inventory (BDI); *n* = 1), low overall eye tracking rates (<80%; *n* = 6) or non-compliance with the instruction to fixate the center of the screen (gaze shifts on 47.5% of hit trials; *n* = 1).

### Study design

Participants attended one session of functional imaging with concomitant eye tracking. Before and after the scanning session, participants filled in questionnaires to assess changes in state anxiety (STAI-S) and positive and negative affect (PANAS) related to the experimental procedure. Furthermore, participants completed versions of the BDI, the State Trait Anxiety Inventory (STAI-T) and the Toronto Alexithymia Scale (TAS-20) to rule out effects of altered mood states or emotional perception.

**Figure 1:**
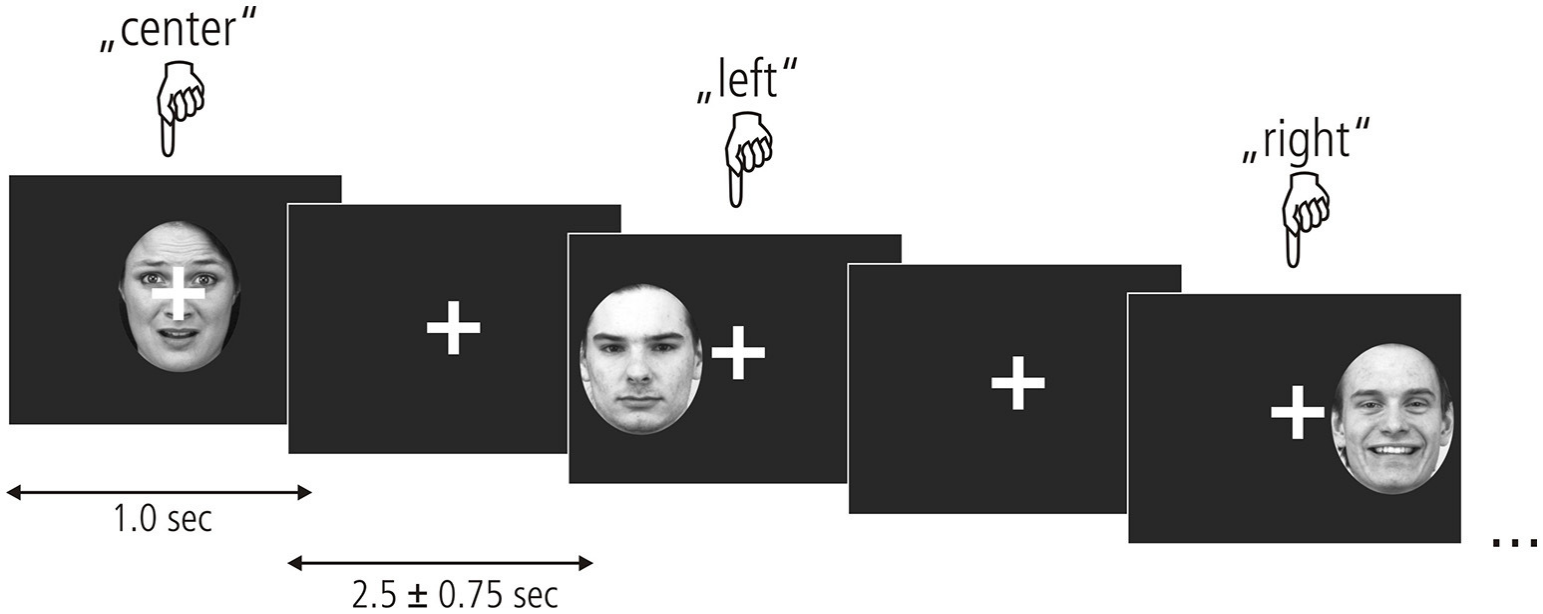
Experimental paradigm. Fearful, neutral, and happy faces from the KDEF picture set (shown here left to right: AF01AFS, AM07NES, and AM21HAS) appeared centrally or in the left/right hemifield for 1 s. A fixation cross appeared after each picture which remained on the screen for 2.5 + –0.75 s. Participants kept their gaze locked to the central crosshairs throughout the experiment and pressed one of three buttons to indicate stimulus location.

To assess the processing of unattended emotional facial expressions in the left, central, or right visual field, we used an event-related design presented in Figure 1. Photographs of fearful, happy and neutral faces taken at a straight angle were taken from the Karolinska Directed Emotional Faces database (KDEF; Lundqvist et al., 1998), transformed into grayscale and clipped by an oval shape to reveal the face. Within each of the nine experimental conditions (3 valences × 3 locations), 22 stimuli were presented containing equal proportions of male and female faces. In total 231 trials were shown, including 33 null events. Stimulus presentation was randomized and different for each participant. Each stimulus appeared for 1 sec followed by an inter-stimulus interval of 2,500 ± 750 ms. A white crosshairs was shown at the center of the screen throughout the entire experiment. Participants were instructed to look only at the fixation cross and not let their gaze be diverted by any of the stimuli. To ensure a continuously high level of attention, participants indicated the position of each stimulus by pressing a response button corresponding to the left (left index), center (right thumb), or right (right index) of the screen. Before functional imaging, participants performed a training session outside of the scanner where they viewed a different set of emotional faces.

By tracking movements of the participants' right eye, we validated compliance with the fixation instruction during scanning. For this purpose, a built-in eye tracker attached to the right ocular of the binocular video goggles (both Nordic NeuroLab, Bergen, Norway) continuously tracked the gaze direction of the participants at a rate of 60 Hz. Button presses were recorded using MR-compatible response grips (Nordic NeuroLab). For stimulus presentation and feedback recording, we used Presentation® (Neurobehavioral Systems, Inc.). Eye movement data were recorded using iView X MRI-SV (SMI, Potsdam, Germany).

### Functional image acquisition

For functional image acquisition we used a 1.5 T Siemens Avanto (Siemens, Erlangen, Germany) equipped with a 12-channel array head coil. By means of a T2*-weighted gradient-echo echo-planar imaging sequence, we acquired 26 axial 3 mm slices with a gap of 40% resulting in an in-plane resolution of 3 × 3 × 4.2 mm3 (TR/TE = 1530/30 ms, FA = 90°, FOV = 192 mm, 64 × 64 matrix). We employed the in-built Siemens iPat mode (integrated parallel acquisition technique) and accelerated the sequence by a factor of 2 using GRAPPA [generalized autocalibrating partially parallel acquisition (Griswold et al., 2002)]. The slices were tilted by about −45° along the line from the lower boundary of the orbitofrontal cortex (BA 11) to the lower boundary of the cerebellar nodule to ameliorate susceptibility artifacts and limit partial volume effects due to tissue borders in the amygdala and temporal cortex (Merboldt et al., 2001; Weiskopf et al., 2006). Subsequent visual inspection of all data sets confirmed good coverage of the amygdala and temporal regions. We collected additional high-resolution anatomical images from each participant using a T1-weighted MPRAGE sequence (176 sagittal slices, 1 mm slice thickness, GRAPPA acceleration factor 2).

## Data analyses

### Behavioral data

Reaction times of hit trials (ms) and error rates (percent) were calculated for each participant and each experimental condition and compared in a repeated-measures ANOVA with factors face valence (fearful, happy, neutral) and face location (left, central, right) using SPSS 17.0 and a significance level of *p* < 0.05 two-sided. We calculated *post-hoc* tests for significant main effects and interactions at a one-sided threshold of *p* < 0.025.

### Eye movement

**Figure 2:**
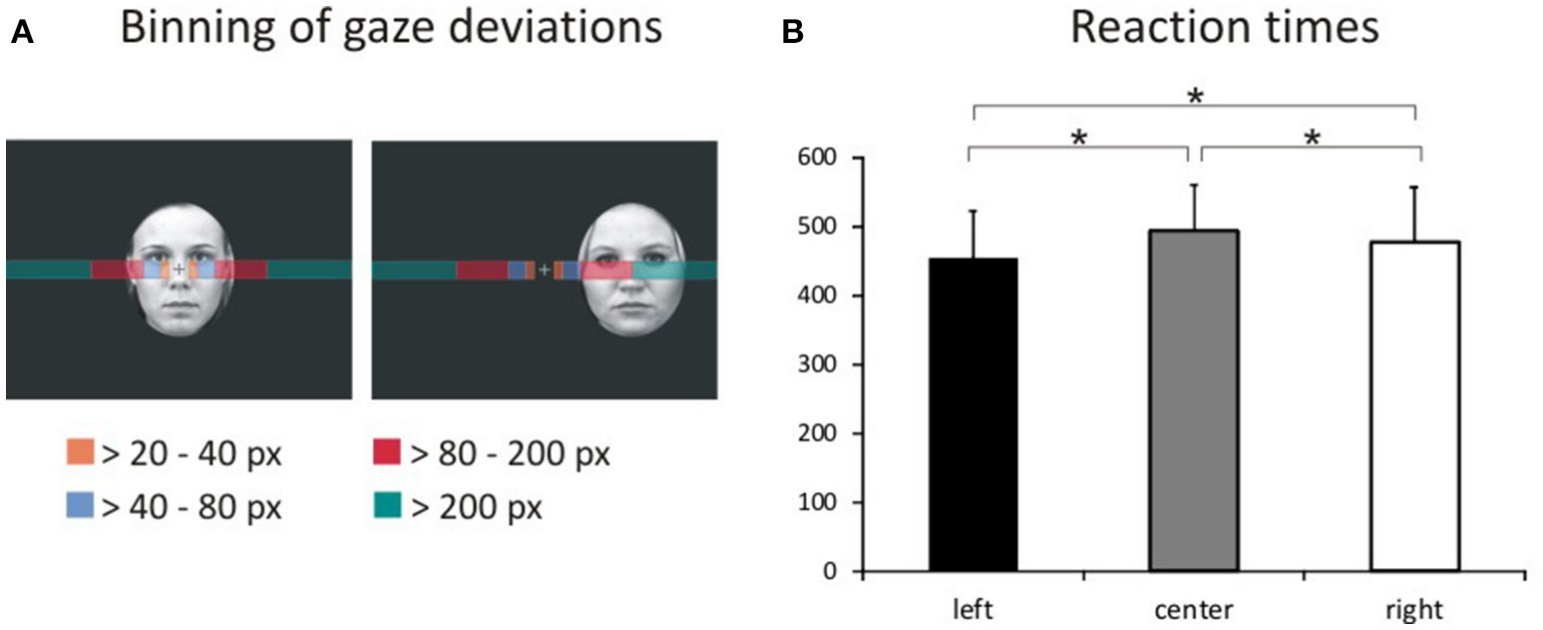
Binning of gaze deviations and visual field bias in behavioral responses. **(A)** We classified gaze deviations in along the horizontal axis in four different bins containing fixations falling within 20–40 pixels, 40–80 pixels, 80–200 pixels, and >200 pixels to the left or right of the central crosshairs. **(B)** Reaction times for pictures presented in the left hemifield, at the center of the screen and in the right hemifield representing the main effect of location.

We assessed task compliance during hit trials using BeGaze 3.0 (SMI) by analyzing means and standard deviations for the frequency of gaze shifts and location of fixations away from the central crosshairs along the horizontal axis (i.e., to the left or right). Frequencies of gaze deviations during hit trials were then binned into four categories (Figure 2A) corresponding to fixations falling into the space between (1) the fixation cross and the center of the inner eye of centrally presented faces (bin 1: >20 to 40 pixels), (2) the center of the inner eye and the inner border of peripherally presented faces (bin 2: >40–80 pixels), (3) the inner border and the center of peripherally presented faces (bin 3: >80 to 200 pixels) and (4) beyond the center of peripherally presented faces (bin 4: >200 pixels). The percentage of hit trials during which the eye tracker registered gaze deviations along the horizontal axis were compared across experimental conditions by means of a repeated-measures ANOVA with the factors gaze direction (left, right), stimulus valence (fearful, happy, neutral), stimulus location (left, central, right), and gaze distance (bin1, bin2, bin3, bin4) using SPSS 17.0 at a two-sided threshold of *p* < 0.05.

### Functional imaging

Before functional data analysis in SPM 8 (http://www.fil.ion.ucl.ac.uk/spm) we discarded the first three volumes of each data set to allow T1 saturation to reach a stable level. Data were realigned to the eighth slice of each volume containing the center of the amygdala as our primary region of interest and then slice timed to reduce variance attributable to motion and differences in slice acquisition time. We coregistered the high-resolution anatomical image to the functional mean image and segmented the coregistered structural image using the gray and white matter tissue probability maps provided in SPM8. Subsequently, we normalized the functional data at a voxel size of 3 × 3 × 3 mm using the normalization parameters from the segmentation procedure. Spatial smoothing with an isotropic Gaussian kernel of 9 mm FWHM reduced high-frequency noise. For each participant, t-statistics for the nine experimental conditions [fearful left (FL), fearful central (FC), fearful right (FR), happy left (HL), happy central (HL), happy right (HR), neutral left (NL), neutral central (NC), neutral right (NR)] were calculated in a first-level fixed effects model corrected for serial autocorrelations (AR1) and low frequency signals (high-pass filter of 128 s). To this end, we modeled boxcar regressors for hit trials from the experimental conditions according to the actual length of the stimuli and convolved these with the canonical hemodynamic response function implemented in SPM8. Regressors of no interest included error trials, as well as the individual realignment parameters. A random effects group level analysis compared interactions of the experimental factors face valence (fearful, happy, neutral) and face location (left, central, right) in a full-factorial within-subject GLM at an intensity threshold of *p* < 0.001 (uncorrected) with an extent cluster threshold of *k* = 4 as calculated by SPM8. Peak coordinates of activated clusters are reported in Table 2 in MNI space along with their corresponding anatomical labels according to the AAL database within the WFU pickatlas 3.0.3 (Tzourio-Mazoyer et al., 2002; Maldjian et al., 2003, 2004).

We extracted contrast estimates for each experimental condition from clusters showing sensitivity to both valence and location and performed *post-hoc* tests on these values for further exploration of the neural activation patterns. In SPSS 17.0 we compared experimental conditions by means of one-sided *t*-Tests for dependent samples at an uncorrected *p* < 0.025 and at *p* < 0.0028 Bonferroni-corrected.

## Results

Participants scored normally with respect to depression (BDI *M* = 2.6, *SD* = 2.4), trait anxiety (STAI-T *M* = 36.0, *SD* = 8.8), and alexithymia (TAS-20 *M* = 37.2, *SD* = 11.7). Positive and negative affect ratings from the PANAS questionnaire did not differ before and after functional imaging [positive affect pre-scan *M* = 22.94, SE = 1.88, post-scan *M* = 23.28, SE = 1.78, *t*_(17)_ = −0.75, *p* = 0.462; negative affect pre-scan *M* = 27.11, SE = 1.13, post-scan *M* = 27.28, SE = 1.28, *t*_(17)_ = 0.85, *p* = 0.852]. Comparing state ratings from the STAI-S questionnaire we found that participants were more anxious before compared to after the scanning session [pre-scan *M* = 50.5, SE = 1.14, post-scan *M* = 48.8, SE = 1.27, *t*_(17)_ = 2.33, *p* = 0.032].

### Behavioral data

The average error rate was 1.49% (*SD* = 1.32). A 3 × 3 ANOVA with factors face valence (afraid, happy, neutral) and face location (left, center, right) yielded a main effect of location [Figure 2B; *F*_(2, 34)_ = 13.4, *p* < 0.001, ${\eta_{\text{p}}}^{2}=0.440$]. *Post-hoc* comparisons of mean RT with respect to location show faster responses to stimuli presented in the left hemifield both compared with stimuli presented centrally [*t*_(53)_ = 7.36, *p* < 0.001] and in the right hemifield [*t*_(53)_ = 4.74, *p* < 0.001]. Moreover, RT to stimuli presented in the right hemifield were faster compared with RT to centrally presented stimuli [*t*_(53)_ = 2.49, *p* = 0.016].

The main effect of face valence was non-significant [*F*_(2, 34)_ = 0.450, *p* = 0.641, ${\eta_{\text{p}}}^{2}=0.026$]. The main effect of face location was not qualified by an interaction between face valence and face location [*F*_(4, 68)_ = 2.22, *p* = 0.076, ${\eta_{\text{p}}}^{2}=0.115$]. Means and standard deviations for error rates and RT for each of the nine experimental conditions are reported in Table 1. Means and standard deviations for the analysis of RT are also given in the Supplementary Material.

**Table 1:** Behavioral and eye tracking results.

					**Gaze deviations along the horizontal axis**															
	**Reaction times**		**Error rates**		**Bin 1 (>20 to 40 pixel)**				**Bin 2 (>40 to 80 pixel)**				**Bin 3 (>80 to 200 pixel)**				**Bin 4 (>200 pixel)**			
	**(ms)**		**(percent)**		**Left**		**Right**		**Left**		**Right**		**Left**		**Right**		**Left**		**Right**	
	**mean**	***SD***	**mean**	***SD***	**mean**	***SD***	**mean**	***SD***	**mean**	***SD***	**mean**	***SD***	**mean**	***SD***	**mean**	***SD***	**mean**	***SD***	**mean**	***SD***
**FEARFUL**																				
Left	459	68	0.25	1.06	0.39	0.78	0.72	1.32	0.28	0.75	0.22	0.65	1.00	1.03	0.17	0.38	0.11	0.32	0.00	0.00
Center	486	67	1.00	1.93	0.50	0.79	0.33	0.84	0.44	0.86	0.06	0.24	0.06	0.24	0.06	0.24	0.00	0.00	0.00	0.00
Right	475	80	2.26	3.21	0.28	0.67	0.11	0.32	0.28	0.57	0.28	0.57	0.33	1.03	0.78	1.35	0.00	0.00	0.11	0.47
**HAPPY**																				
Left	452	76	0.00	0.00	0.33	0.97	0.11	0.32	0.28	0.57	0.50	0.92	0.83	1.72	0.06	0.24	0.17	0.38	0.00	0.00
Center	491	58	1.76	2.75	0.67	1.33	0.22	0.55	0.28	0.83	0.44	0.70	0.00	0.00	0.11	0.32	0.00	0.00	0.00	0.00
Right	484	86	3.03	4.13	0.39	0.98	0.22	0.73	0.50	0.92	0.39	0.61	0.28	0.83	1.00	0.91	0.00	0.00	0.17	0.51
**NEUTRAL**																				
Left	452	64	0.00	0.00	0.50	1.15	0.28	0.57	0.39	0.70	0.67	0.91	0.61	1.04	0.11	0.32	0.17	0.38	0.00	0.00
Center	503	63	1.26	2.60	0.33	0.77	0.17	0.51	0.39	0.85	0.33	0.84	0.00	0.00	0.00	0.00	0.00	0.00	0.06	0.24
Right	476	85	1.00	1.93	0.33	0.69	0.33	0.84	0.44	0.98	0.22	0.55	0.06	0.24	0.94	1.06	0.00	0.00	0.11	0.47

*Mean values and standard deviations (SD) for the nine experimental conditions reporting reaction times (ms) for correct trials, error rates (percent), and percent tracked hit trials with gaze deviations to the left or right corresponding to the bins displayed in Figure 2A*.

**Table 2:** Brain regions showing interaction effects of facial valence and stimulus location.

**Region**	**Right/Left**	**MNI-coordinates**			**Cluster size (voxels)**	***Z*- score**
		**x**	**y**	**z**		
**INTERACTION “FACE LOCATION X FACE VALENCE”**						
Temporal Pole	L	−45	8	−32	15	4.36
Middle temporal gyrus	L	−57	−40	−5	5	3.56
Amygdala	R	15	−4	−26	14	3.65
	R	12	2	−20		3.27
Putamen	R	33	−19	1	4	3.63
Superior frontal gyrus (frontal eye field)	R	24	38	49	5	3.35

*The whole-brain analysis was thresholded at an uncorrected p < 0.001 with a cluster extent threshold of k = 4 voxels. FC, fearful center; FL, fearful left; FR, fearful right; HC, happy center; HL, happy left; HR, happy right; NC, neutral center; NL, neutral left; NR, neutral right*.

### Eye movement data

The average tracking rate of hit trials was 97.4% (*SD* = 5.3). The average percentage of trials with gaze deviations away from the central crosshairs was 10.8% (*SD* = 11.8). Eye movement data were also analyzed using a within-subjects ANOVA with factors gaze direction (left, right), face location (left, center, right), face valence (afraid, happy, neutral) and gaze distance (bin1, bin2, bin3, bin4). Degrees of freedom were corrected using Greenhouse-Geisser estimates of sphericity when Mauchly's test indicated that the assumption of sphericity had been violated. Asterisks mark the respective contrasts.

This analysis revealed a main effect of face location [*F*_(2, 34)_ = 4.81, *p* = 0.014, ${\eta_{\text{p}}}^{2}=0.221$]. More gaze shifts occurred during trials with peripherally presented stimuli compared with centrally presented stimuli (left vs. center *p* = 0.015; right vs. center *p* = 0.037).

We also found a main effect of gaze distance [*F*_(3, 51)_ = 3.95, *p* = 0.013, ${\eta_{\text{p}}}^{2}=0.168$]. More gaze shifts fell into the first three bins compared with the most distal bin (bin 1 vs. 4 *p* = 0.030, bin 2 vs. 3 *p* = 0.010, bin 3 vs. 4 *p* = 0.002).

These effects were qualified by two-way interactions of face location × gaze distance [*F*_(2.7, 45.2)_ = 5.01, *p* = 0.006^*^, ${\eta_{\text{p}}}^{2}=0.228$] and gaze direction × face location [*F*_(1.4, 23.8)_ = 9.82, *p* = 0.002^*^, ${\eta_{\text{p}}}^{2}=0.366$], as well as by a three-way interaction of gaze direction × face location × gaze distance [*F*_(2.4, 39.9)_ = 7.29, *p* = 0.001^*^, ${\eta_{\text{p}}}^{2}=0.300$].

There was no significant main effect or interaction with stimulus valence (all *F* ≤ 1.78, *p* ≥ 0.150, ${\eta_{\text{p}}}^{2}\leq 0.093$) or any other main effects and interactions (all *F* ≤ 0.39, *p* ≥ 0.668, ${\eta_{\text{p}}}^{2}$ ≤ 0.023). All means and standard deviations for gaze deviations along the horizontal axis are reported in Table 1. Means and standard deviations for the analysis of eye movements are also given in the Supplementary Material.

### Functional imaging

**Figure 3:**
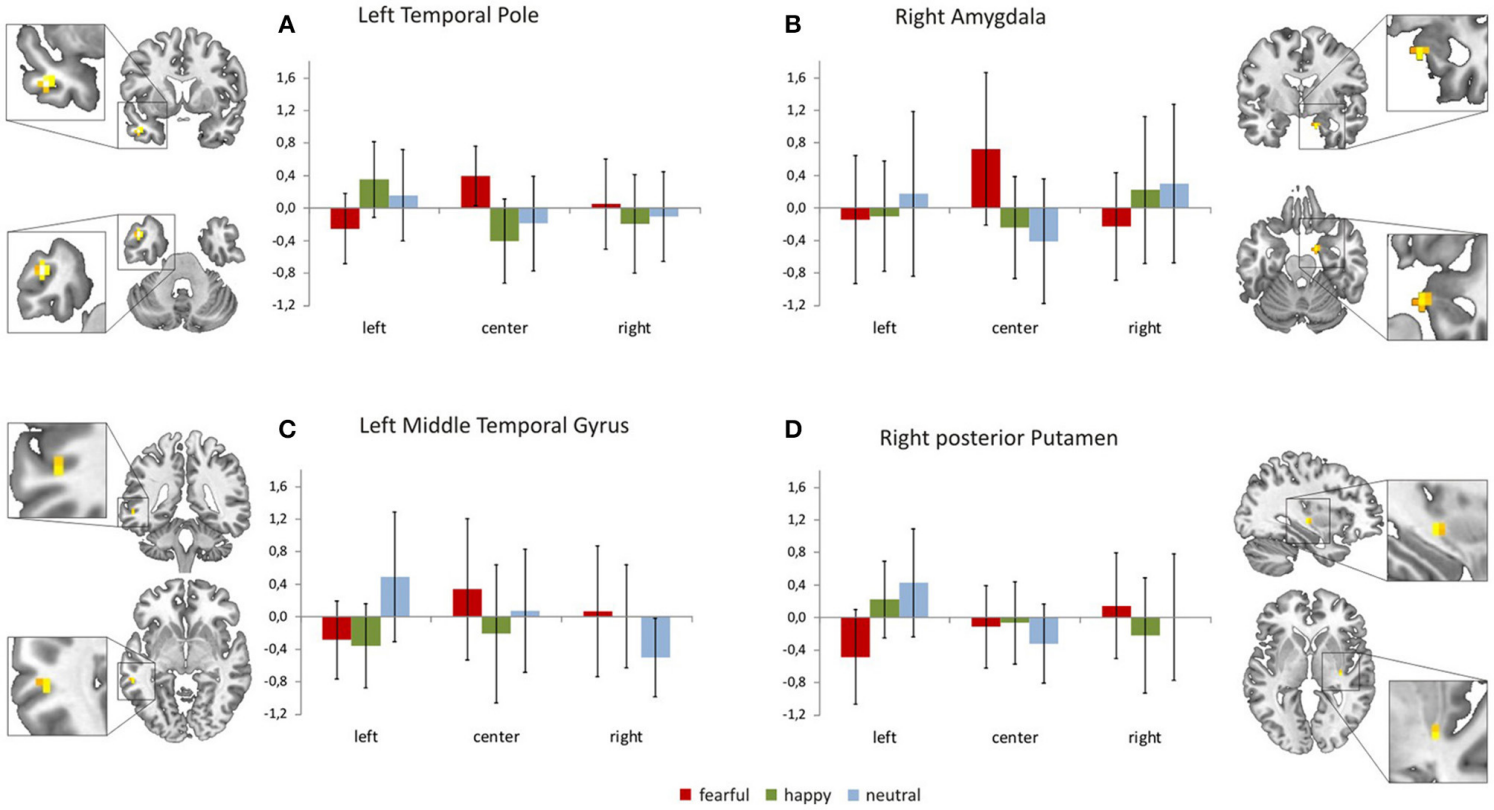
Brain regions sensitive to both face valence and stimulus location. The left temporal pole **(A)**, the right amygdala **(B)**, the left middle temporal gyrus **(C)**, the right posterior putamen **(D)**, and the right superior frontal gyrus (not shown, see Supplementary Figure 1) exhibit differential activation patterns with respect to both stimulus location and face valence (mean contrast estimates and 90% confidence intervals from each cluster showing an interaction of location × valence, *p* < 0.001 uncorrected, cluster extent threshold *k* ≥ 4).

Focussing on the interaction of face location × face valence on the whole brain level, we found activation in the left temporal pole, left middle temporal gyrus, right amygdala, right posterior putamen and the right superior frontal gyrus (uncorrected *p* < 0.001, *k* = 4). In a *post-hoc* analysis we compared mean contrast estimates from the activated clusters using one-sided *t*-Tests with a statistical threshold of *p* < 0.025 (comparisons that are also significant at a Bonferroni-corrected *p* < 0.0028 are marked by asterisks; see Figure 3). In this analysis, we found a subset of the above-mentioned regions to be sensitive to the central presentation of fearful faces. This subset includes the left temporal pole [Figure 3A; FC>FL: *t*_(17)_ = 4.91, *p* < 0.0001^*^], the right amygdala [Figure 3B; FC>FR: *t*_(17)_ = 3.36, *p* = 0.0019^*^; FC>FL: *t*_(17)_ = 2.75, *p* = 0.0069] and the left middle temporal gyrus [Figure 3C; FC>FL: *t*_(17)_ = 2.70, *p* = 0.0075]. The following regions responded to neutral faces presented in the periphery, especially in the left hemifield: the right amygdala [NL>NC: *t*_(17)_ = 3.08, *p* = 0.0034; NR>NC: *t*_(17)_ = 2.12, *p* = 0.024], the left middle temporal gyrus [NL>NR: *t*_(19)_ = 5.01, *p* < 0.0001^*^], the right posterior putamen [Figure 3D; NL>NC: *t*_(17)_ = 3.88, *p* = 0.0006^*^; NL>NR: *t*_(17)_ = 2.34, *p* = 0.0159], and the right superior frontal gyrus [Supplementary Figure 1; NL>NC: *t*_(17)_ = 3.02, *p* = 0.0039; NL>NR: *t*_(17)_ = 2.11, *p* = 0.025]. Happy faces presented in the left hemifield activated the right posterior putamen [HL>HR: *t*_(17)_ = 2.27, *p* = 0.0183] and the left temporal pole [HL>HC: *t*_(17)_ = 4.67, *p* = 0.0001^*^; HL>HR: *t*_(17)_ = 3.86, *p* = 0.0006^*^]. Main effects of face location and face valence are shown in the Supplementary Material.

## Discussion

The central goal of the present study was to replicate behavioral and neural response patterns indicating a visual field bias for face processing in our female sample. In particular, we were interested in identifying brain regions that are specifically sensitive both to the (attended) location and the (unattended) valence of emotional facial expressions.

The overall tracking rate of eye movements was excellent. Participants accurately performed the task as indicated by low error rates and a high compliance with the instruction to keep their gaze locked on the central crosshairs.

The percentage of hit trials during which participants shifted their gaze along the horizontal axis varied with respect to the location of stimuli and the distance from the central crosshairs. Gaze shifts were mostly directed at peripherally presented pictures. The greatest percentage of gaze shifts occurred during peripherally presented faces and fell into the range between 80 and 200 pixels away from the center of the screen. In the range between 20 and 80 pixels away from the central crosshairs, gaze shifts to the left and right occurred for all stimulus locations. In the range from 80 pixels upwards, gaze shifts were directed at peripheral stimuli.

We found a visual field bias on the behavioral level: participants responded faster to pictures presented in the left hemifield compared with faces presented in the right hemifield or at the center of the screen. However, behavioral responses did not differ with respect to stimulus valence.

Our present results also indicate a complementary visual field bias in neural processing that is valence sensitive. The neural activation pattern in response to fearful, neutral and happy faces in brain regions commonly associated with face processing varies with respect to stimulus location (Figures 3A–D, Supplementary Figure 1). The left temporal pole, the left middle temporal gyrus and the right amygdala showed a sensitivity to both valence category and stimulus location as they responded to fearful faces presented at the center of the screen. Processing of peripherally presented neutral faces (especially when they appeared in the left hemifield) involved the right amygdala, the left middle temporal gyrus, the right posterior putamen and the right superior frontal gyrus (frontal eye field). Happy face stimuli presented outside foveal perception elicited responses in the left temporal pole and the right posterior putamen; again, these regions were particularly responsive to happy faces appearing in the left hemifield.

### Unattended threat signals

In the present study, the right amygdala was sensitive to centrally presented fearful faces and to neutral faces presented in the left hemifield. The amygdala has a well-documented role in the processing of emotional and neutral facial expressions (Haxby et al., 2002; Platek et al., 2006; Olson et al., 2007) even when they are unconsciously perceived (Vuilleumier, 2005). The ability to rapidly detect potentially relevant stimuli signaling the need for withdrawal are, at least in part, related to a subcortical circuit comprising of the amygdala, the superior colliculus and the pulvinar nucleus of the thalamus (Morris et al., 1999; LeDoux, 2000, 2007; Tamietto and de Gelder, 2010). Behavioral studies of unconscious emotional processing using masked stimuli also revealed enhanced autonomic responses to fearful faces presented in the left visual field compared with fearful faces presented in the right visual field or neutral faces presented in either field (Kimura et al., 2004).

Several authors found different neural correlates for explicit vs. implicit threat processing (Critchley et al., 2000; Habel et al., 2007; Tamietto and de Gelder, 2010; Almeida et al., 2013). While the brainstem and temporal regions such as the superior temporal gyrus were especially responsive during explicit tasks (Critchley et al., 2000), the amygdala was involved in both explicit (Habel et al., 2007) and implicit tasks (Critchley et al., 2000).

Complex perceptual inputs and visceral responses are integrated e.g., in the temporal pole (Olson et al., 2007). The present results corroborate the proposed involvement of the amygdala/temporal pole complex in the perception of (potential) threat signals, such as centrally perceived fearful faces and peripherally perceived neutral faces.

### Stimulus location

Studies directly comparing central and peripheral vision point to a central bias for both human and animal faces (Liu and Ioannides, 2010; Morawetz et al., 2010; Almeida et al., 2013). As defined by Strasburger et al. (2011) central/foveal vision refers to the perception of objects within 2° of eccentricity, while the perception of objects presented beyond 2° of eccentricity is classified as peripheral vision. In a functional imaging study Almeida et al. (2013) systematically manipulated spatial location (central or peripheral) and stimulus type (face, real or fake snake shape) as a main outcome measure while using both implicit and explicit threat tasks. This study found foveal representations in subcortical structures such as the amygdala, the pulvinar nucleus of the thalamus and the superior colliculus. As these regions played distinct roles in the central and peripheral processing of snake shapes the authors concluded that there are multiple phylogenetic fingerprints in the subcortical responses to fear-relevant stimuli. In the present study, a distributed network including the amygdala, posterior putamen, temporal cortex and superior frontal cortex was sensitive to face valence of stimuli presented at foveal, and peripheral locations. These findings are in line with the view that stimuli such as faces demand detailed processing whereas peripheral object processing is based on a rather coarse identification (Kanwisher, 2001; Levy et al., 2001). In this context, the amygdala receives direct input from ventral areas that show a known bias toward foveal (central) input.

Behavioral studies suggest that parafoveal and peripheral locations entail relatively sound performance in stimulus recognition and categorization (Malach et al., 2002; Calvo and Lang, 2005). This is especially true for faces which can be efficiently processed and detected when presented outside of the fovea (Carretié, 2014). Investigating how the amount of stimulus eccentricity influences behavioral and neural responses, Rigoulot et al. (2011) created an experimental setup comparing the categorization of fearful and neutral faces at four peripheral locations (15° and 30° to the right and left) in a female sample. Less eccentricity as well as more negative emotional valence detrimentally influenced performance in that reaction times were shorter for fearful faces and for faces presented at 15° eccentricity. Fearful faces also entailed greater N170 amplitudes, even at far eccentric locations, further corroborating the notion that the preferential neural coding of fearful expressions persists even in far peripheral vision.

The behavioral substrate of a visual field bias in the present study is in line with split-field and lesion studies reporting a left visual field superiority for the perception of emotional facial expressions in general (e.g., Mandal and Singh, 1990; Mandal et al., 1991). Our results corroborate the view that the evaluation of emotional facial expressions happens automatically even when this feature is task-irrelevant (Pessoa, 2008). This automatic evaluation does not necessarily seem to entail a detrimental effect on task performance as suggested by the lack of valence-specific effects on both response times and eye movements in the present study.

### Valence specificity

In addition to the neural responses to threat-related stimuli, we also found specific responses to neutral and/or approach-related stimuli. When faces appeared in the left hemifield, the left temporal pole and the right posterior putamen responded to happy faces; the left middle temporal gyrus, the right amygdala, the right posterior putamen and the right superior frontal gyrus responded to neutral faces. These results complement previous research finding that salient face stimuli activate the middle temporal gyrus (Haxby et al., 2002; Platek et al., 2006), putamen (Leveroni et al., 2000; Fu et al., 2007) and superior frontal gyrus (Ebner et al., 2012). Our results also corroborate previous evidence regarding the endogenous (top-down) attention capturing quality of emotional stimulation when subjects perceive emotional stimulation outside their attention focus (Carretié, 2014).

Our functional imaging results yielded an activation pattern that is different from the “valence hypothesis” stating that the right hemisphere is specialized in processing negative emotions whereas the left hemisphere is specialized in processing positive emotions (Davidson, 1992, 1995). In humans, lateralization seems to be quite strict at the primary sensory level (e.g., contralateral processing of visual input in the primary visual cortex) but less obvious when it comes to more complex cognitive functions such as face processing (see Phan et al., 2002; Murphy et al., 2003; Wager et al., 2003 for review). Sung et al. (2011) suggest that a static circuit similar to passive information filtering is responsible for the involvement of low-level visual areas. Depending on the task, higher areas might dynamically modulate these neural circuits, as a right hemispheric (i.e., left visual field) superiority for facial processing differs with respect to stimulus content such as the orientation of faces in the picture (upright or inverted). Based on others' as well as our own previous findings (Preibisch et al., 2009) we suggest that functional hemispheric specialization in face processing does not necessarily require lateralization to one hemisphere. Rather, preferred pathways for emotional contents are required which may depend, among other factors, on the side of input and the emotional content of the stimuli. This claim is supported by the fact that we found the network that processes potential threat signals to be sensitive to both valence category and stimulus location. A set of regions including the right amygdala, left temporal cortex, right posterior putamen and right superior prefrontal cortex responded to centrally presented fearful faces and/or peripherally presented happy and neutral faces especially in the left visual hemifield suggesting a neural substrate of a visual field bias (Asthana and Mandal, 2001; Vauclair and Donnot, 2005; Proverbio et al., 2006).

## Conclusion

Taken together, the pattern of findings observed in the present study suggests the presence of a sensitive functional network underlying both central and peripheral perception of unattended emotional facial expressions. This network prominently involves the amygdala, temporal pole and posterior putamen whose role in face processing is backed by a large body of literature (Ekman, 1993; Adolphs et al., 1994; Olson et al., 2007; Pessoa, 2008). The specifics of behavioral and neural responses seem to differ with respect to the location, and in the case of neural responses also the valence, of facial expressions (Killgore and Yurgelun-Todd, 2001; Sabatinelli et al., 2004). Although, we cannot claim gender specificity as no formal statistical comparison was calculated, the results of the present study are overlapping yet distinct from our previous findings on a neural visual field bias in men (Preibisch et al., 2009). Future research in this direction thus warrants a direct comparison between male and female subjects within the same study to clarify how the central vs. peripheral perception of unattended emotional facial expressions relates to gender.

## Availability of data and materials

The datasets used and/or analyzed during the current study are available in the Mendeley Data repository, https://doi.org/10.17632/fpzp4df5nc.2

## Author contributions

DW designed the study based on a previous study designed by CP. DW conducted the experiments and analyzed the data. DW drafted the manuscript, CP and HL provided critical revisions.

### Conflict of interest statement

The authors declare that the research was conducted in the absence of any commercial or financial relationships that could be construed as a potential conflict of interest.

## Supplementary material

Supplementary Figure 1 Mean contrast estimates and 90% confidence intervals from the right superior frontal gyrus showing an interaction of location × valence, *p* < 0.001 uncorrected, cluster extent threshold *k* ≥ 4.

Supplementary Figure 2 Brain regions showing a main effect of valence, *p* < 0.001 uncorrected, cluster extent threshold *k* ≥ 4.

Supplementary Figure 3 Brain regions showing a main effect of location, *p* < 0.05 FEW-corrected.

Supplementary Table 1 Means, standard deviations, and results from the ANOVA on reaction times.

Supplementary Table 2 Means, standard deviations, and results from the ANOVA on eye movements.
