# Supplementary figures and images for: Processing of Unattended Emotional Facial Expressions: Correlates of Visual Field Bias in Women
Source: Front Neurosci. 2017 Aug 14;11:443. doi: 10.3389/fnins.2017.00443 (PMC5557747; doi:10.3389/fnins.2017.00443)

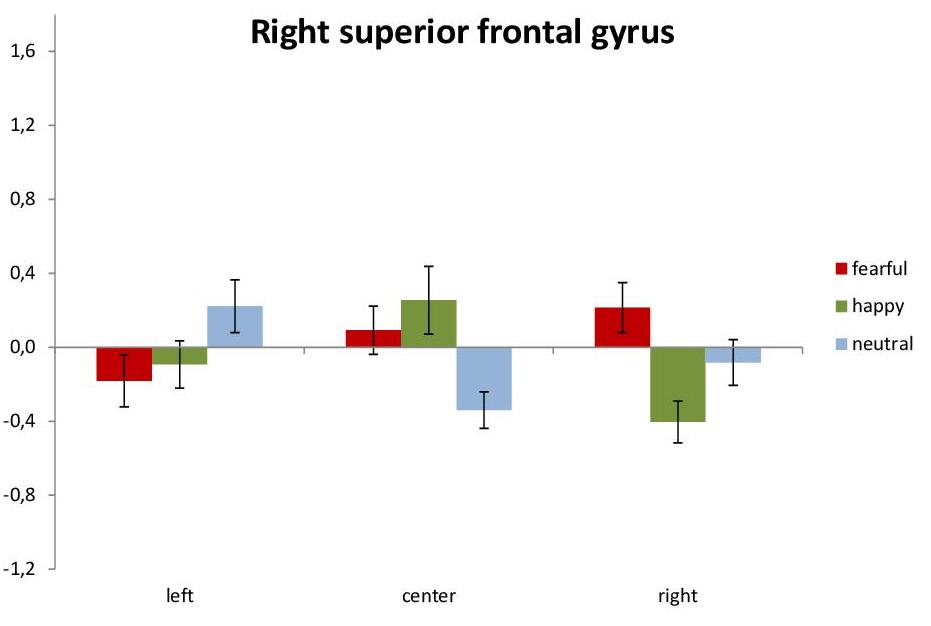

Supplement: Supplementary Figure 1 — Mean contrast estimates and 90% confidence intervals from the right superior frontal gyrus showing an interaction of location × valence, p < 0.001 uncorrected, cluster extent threshold k ≥ 4. [file Image1.JPEG]

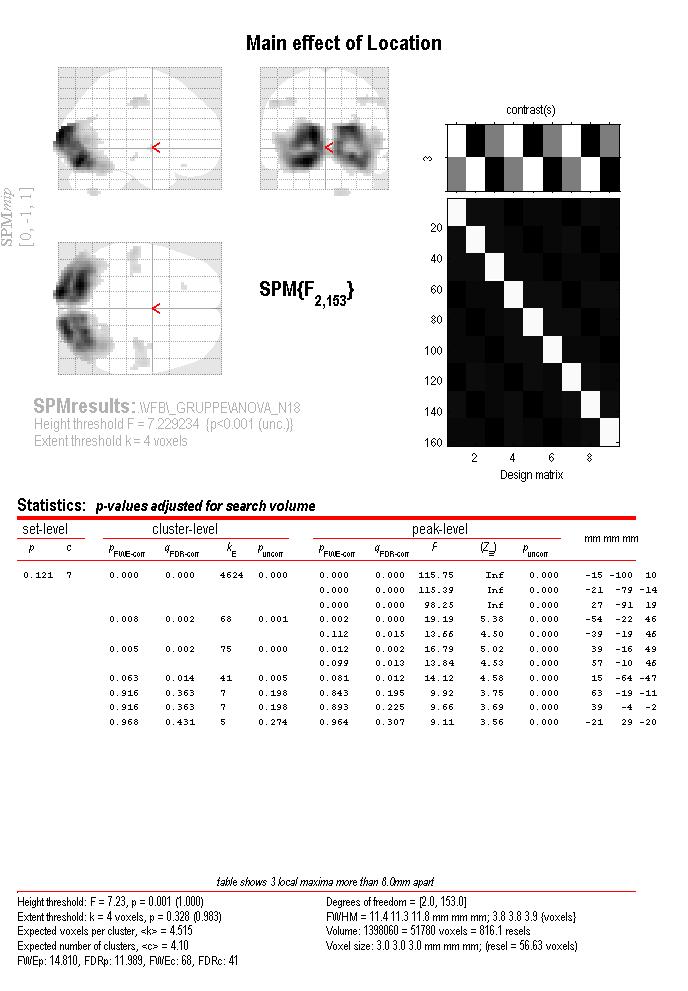

Supplement: Supplementary Figure 2 — Brain regions showing a main effect of valence, p < 0.001 uncorrected, cluster extent threshold k ≥ 4. [file Image2.JPEG]

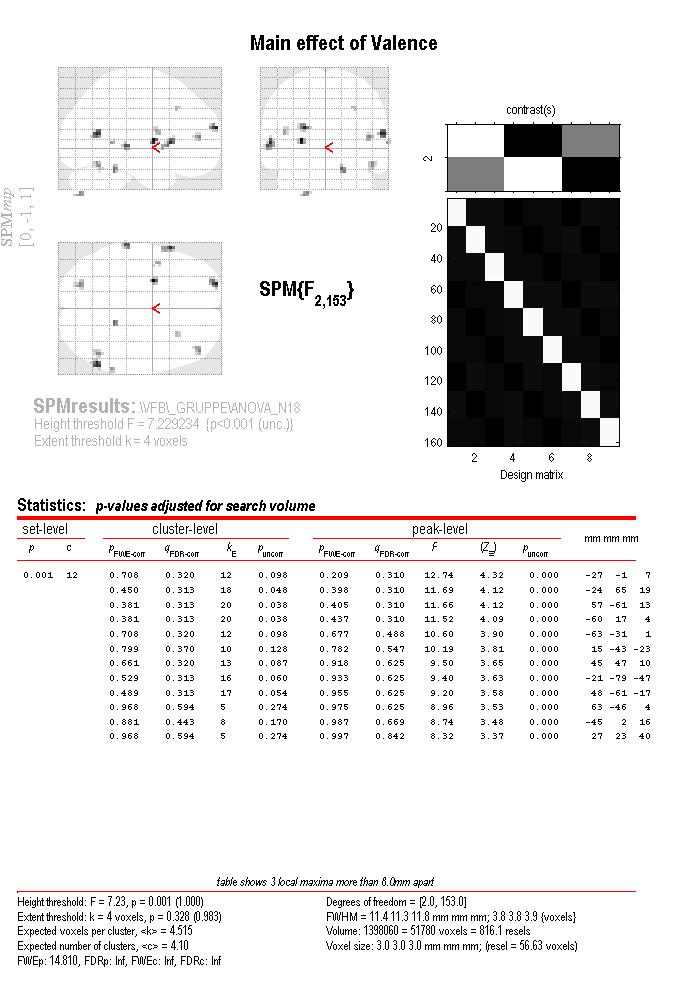

Supplement: Supplementary Figure 3 — Brain regions showing a main effect of location, p < 0.05 FEW-corrected. [file Image3.JPEG]
